# Die Bedeutung schulischer Gesundheitsförderung für die Erhöhung gesundheitlicher Chancengleichheit

**DOI:** 10.1007/s00103-022-03551-w

**Published:** 2022-06-03

**Authors:** Irene Moor, Janis Bieber, Liska Niederschuh, Kristina Winter

**Affiliations:** Institut für Medizinische Soziologie, Martin-Luther-Universität Halle-Wittenberg, Magdeburger Str. 8, 06112 Halle/Saale, Deutschland

**Keywords:** Gesundheitliche Ungleichheiten, Schule, Kinder und Jugendliche, Sozioökonomischer Status, Setting-Ansatz, Health inequalities, Health promoting school, Children and adolescents, Socioeconomic status, School health

## Abstract

Sozioökonomisch bedingte Ungleichheiten in der Gesundheit sind ein wichtiges Public-Health-Handlungsfeld und deren Reduzierung eines ihrer wichtigsten Ziele. Bislang ist es jedoch kaum gelungen, gesundheitliche Ungleichheiten zu verringern, was zugleich auch auf ein großes Forschungsdefizit hinweist. Auch im Kindes- und Jugendalter lassen sich bereits Ungleichheiten in der Gesundheit und dem Gesundheitsverhalten feststellen, wobei jene mit einem niedrigen sozioökonomischen Status verglichen mit sozial privilegierteren Gleichaltrigen oftmals mehr Risikofaktoren und gleichzeitig weniger Ressourcen aufweisen. Obwohl Gesundheitsförderung auf gesundheitliche Chancengleichheit abzielt, berücksichtigen Interventionen nur selten den sozioökonomischen Status und können entsprechend wenig Evidenz über sozioökonomisch differenzierte Interventionseffekte liefern. Wie kann es daher gelingen, allen Heranwachsenden die gleichen Chancen auf ein gesundes Aufwachsen zu ermöglichen?

Um der Beantwortung dieser Frage näherzukommen, ist es zum einen das Ziel des Beitrags, einen Überblick über gesundheitliche Ungleichheiten im Kindes- und Jugendalter zu geben und die Rolle von Gesundheitsförderung sowie aktuelle Forschungsdefizite in diesem Zusammenhang aufzuzeigen. Zum anderen soll die Bedeutung der Schule und schulischer Gesundheitsförderungsmaßnahmen bei der Verringerung gesundheitlicher Ungleichheiten und der Erhöhung gesundheitlicher Chancengleichheit herausgestellt werden. Die Schule ist nicht nur ein Ort, an dem Heranwachsende unabhängig von ihrer sozialen Herkunft stets erreicht werden können, sondern hat das Potenzial, gesundheitliche Ungleichheiten sowohl zu verschärfen als auch zu reduzieren.

## Abstract (en)

Socioeconomic health inequalities are a major field of action in public health and tackling them is one of its main goals. However, there has been little success in reducing these health inequalities. This points to a large research deficit. Inequalities in health are already evident in childhood and adolescence, showing that those with a low socioeconomic background have more risk factors and at the same time fewer resources than their socially more privileged peers. Although health promotion aims at achieving equity in health, interventions rarely consider the socioeconomic status and can therefore provide only little evidence regarding socioeconomically differentiated intervention effects. How can we ensure that all children and young people have equal chances to grow up healthy?

In order to come closer to answering this question, it is the aim of this article to give an overview of health inequalities in childhood and adolescence and to highlight the contribution of school and school-based health promotion interventions for tackling health inequalities and increasing equity in health. School is not only a place where adolescents can always be reached, regardless of their socioeconomic background, it has the potential to both exacerbate and reduce health inequalities.

## Einleitung

### Gesundheitliche Ungleichheiten im Kindes- und Jugendalter

Kinder und Jugendliche stellen eine zentrale Zielgruppe der Prävention und Gesundheitsförderung dar, da in dieser Lebensphase die Weichen ihrer zukünftigen Gesundheit gestellt werden. Gesundheitliche Beeinträchtigungen machen sich nicht nur im aktuellen Gesundheitszustand Jugendlicher bemerkbar, sondern haben häufig langfristige negative gesundheitliche Auswirkungen in Form von höheren Risiken für Erkrankungen im Erwachsenenalter [1]. Auch die gesundheitsbezogenen Einstellungen sowie das Gesundheits- bzw. Risikoverhalten werden in der Adoleszenz ausgebildet und häufig bis in das Erwachsenenalter hineingetragen. Zur Verfügung stehende Ressourcen (Schutzfaktoren) oder auch Risikofaktoren beeinflussen die Gesundheit und bleiben oftmals über den Lebenslauf bestehen [1].

Während der Gesundheitszustand und gesundheitsrelevante Verhaltensweisen von Kindern und Jugendlichen in Deutschland und vielen anderen europäischen Ländern insgesamt als gut bezeichnet werden können und sich in den letzten Jahrzehnten verbessert haben [2, 3], steht dieser positiven Entwicklung die Tatsache entgegen, dass die gesundheitliche Lage junger Menschen eng an ihre soziale Herkunft geknüpft ist und Heranwachsende mit niedrigem sozioökonomischen Status (SES) deutlich häufiger in ihrer Gesundheit beeinträchtigt sowie höheren Risiken ausgesetzt sind und gleichzeitig über weniger protektive Ressourcen verfügen [2, 3]. Beispielsweise zeigen sich eklatante sozioökonomische Ungleichheiten in der subjektiven und psychischen Gesundheit [4, 5] bzw. bei emotionalen und verhaltensbezogenen Problemen [6], in der Lebenszufriedenheit [5, 7], im Substanzkonsum [8, 9] oder auch im Übergewicht [10]. Ähnliche Befunde zeigen sich auch unter Berücksichtigung anderer Ungleichheitsmerkmale wie der Bildung. Welche Schulform das Kind besucht, ist stark an die elterliche Bildung geknüpft und Schülerinnen und Schüler, die nicht das Gymnasium besuchen, weisen insgesamt eine schlechtere Gesundheit und ein ungünstigeres Gesundheitsverhalten auf [11]. Zeitliche Trends verweisen darauf, dass gesundheitliche Ungleichheiten im Jugendalter in den vergangenen Jahrzehnten überwiegend persistent blieben oder sich teilweise sogar vergrößert haben [12, 13].

Sozioökonomisch bedingte Ungleichheiten im Jugendalter beeinflussen demzufolge maßgeblich gegenwärtige wie auch zukünftige Lebens‑, Bildungs- und Gesundheitschancen im Erwachsenenalter. Die Reduktion dieser gesundheitlichen Ungleichheiten sollte daher in Politik, Praxis und Wissenschaft in den Mittelpunkt rücken [14, 15]. Die Frage ist jedoch, *wie* Politik und Praxis diesen Problemlagen begegnen (sollten).

Das Ziel des Beitrags ist es, die Bedeutung der Schule bzw. schulischen Gesundheitsförderungsmaßnahmen zur Verringerung gesundheitlicher Ungleichheiten im Kindes- und Jugendalter herauszustellen sowie auf aktuelle Forschungsdefizite hinzuweisen. Im Folgenden werden zunächst die Relevanz gesundheitlicher Ungleichheiten für Maßnahmen der Gesundheitsförderung und Prävention sowie mögliche Auswirkungen dieser Interventionen auf gesundheitliche Ungleichheiten beleuchtet. Anschließend werden die Schule und ihre Potenziale hinsichtlich der Verringerung gesundheitlicher Ungleichheiten und Erhöhung gesundheitlicher Chancengleichheit diskutiert. Der Beitrag widmet sich möglichen Ansatzpunkten und der gegenwärtigen Situation in Deutschland und schließt mit der Diskussion ab, was Schule leisten kann und wie Maßnahmen gestaltet werden müssen, um erfolgreich zu sein.

## Die Bedeutung gesundheitlicher Ungleichheiten für Gesundheitsförderung und Prävention

Die Ottawa-Charta der Weltgesundheitsorganisation (WHO) zur Gesundheitsförderung (1986) beschreibt diese als „… einen Prozess, allen Menschen ein höheres Maß an Selbstbestimmung über ihre Gesundheit zu ermöglichen und sie damit zur Stärkung ihrer Gesundheit zu befähigen“ [16]. Weiter heißt es bezüglich gesundheitlicher Ungleichheiten: „Gesundheitsförderung ist auf *Chancengleichheit* auf dem Gebiet der Gesundheit gerichtet. Gesundheitsförderndes Handeln bemüht sich darum, bestehende soziale Unterschiede des Gesundheitszustandes zu verringern sowie gleiche Möglichkeiten und Voraussetzungen zu schaffen, damit alle Menschen befähigt werden, ihr größtmögliches Gesundheitspotential zu verwirklichen“ [16].

Was wird unter gesundheitliche Ungleichheiten und gesundheitliche Chancengleichheit verstanden? Im englischsprachigen Raum wird zwischen „health inequalities“ und „health inequities“ unterschieden [17]. Während Erstere gesundheitliche Ungleichheiten im Sinne von Unterschieden meinen (z. B. aufgrund von biologischen Unterschieden, wie etwa des Alters), bezeichnet der zweite Begriff gesundheitliche Ungerechtigkeiten, d. h. gesundheitliche Unterschiede aufgrund von sozial bedingten Umständen (z. B. den sozial differenten Lebensbedingungen), welche als vermeidbar und damit auch als ungerecht empfunden werden [18]. Die Begrifflichkeiten werden jedoch meist synonym verwendet, so sind mit gesundheitlichen Ungleichheiten überwiegend Ungerechtigkeiten gemeint.

Obwohl gesundheitliche Chancengleichheit bzw. Chancengerechtigkeit auch oftmals synonym verwendet wird, unterscheidet sich hier ebenfalls die Bedeutung. Nach Whitehead [18] bezieht sich Gleichheit („equality“) auf die Ergebnisse (also das Ziel) und Gerechtigkeit („equity“) auf die Handlungsebene (den Prozess, der zum Ziel führt). Gesundheitliche Chancengleichheit ist folglich als ein gesundheitspolitisches Ziel anzusehen, welches allen Menschen die gleichen Möglichkeiten zur Entwicklung, Erhaltung und Wiederherstellung ihrer Gesundheit ermöglichen soll. Da jedoch nicht alle die gleichen Ausgangsbedingungen haben, bedeutet gesundheitliche Chancengerechtigkeit die notwendigen Bedingungen dafür zu schaffen und beschreibt damit das operative Umsetzungsprinzip [18].

Nach diesem Prinzip müsste das Ziel der WHO der gesundheitlichen Chancengleichheit also die Möglichkeiten einräumen, gesundheitliche Chancengerechtigkeit zu erwirken. Doch obwohl laut der WHO Gesundheitsförderung per Definition explizit auf gesundheitliche Chancengleichheit abzielt, scheint dieses Ziel in vielen Maßnahmen zur Gesundheitsförderung bisher kaum umgesetzt. Ein möglicher Grund könnte darin bestehen, dass nur wenig empirisch gesichertes Wissen darüber besteht, welche Arten gesundheitsfördernder und präventiver Maßnahmen tatsächlich zu einer Verringerung gesundheitlicher Ungleichheiten beitragen können und welche spezifischen Wirkmechanismen ihnen zugrunde liegen. Whitehead [19] wies auf diese Forschungslücke hin und forderte, dass Interventionen der Prävention und Gesundheitsförderung stets auch Ungleichheitsaspekte berücksichtigen sollten. Dies erfolgt bisher jedoch nur unzureichend, wie u. a. 2 systematische Reviews zu schulischen Interventionen bzw. Interventionen im Jugendalter zeigten [20, 21]. Sowohl die Kommission Sozialer Determinanten der Gesundheit als auch die WHO setzen sich für eine bessere Evidenzgrundlage ein, um Aussagen darüber treffen zu können, ob und wie sich Interventionen auf gesundheitliche Ungleichheiten auswirken. Bisweilen ist es jedoch noch weitgehend unklar, wie gesundheitlichen Ungleichheiten am effektivsten begegnet werden kann und inwiefern bisherige Maßnahmen alle Teilnehmenden in gleicher Weise erreichen [21].

Herausforderungen der Gesundheitsförderung mit und für sozial benachteiligte Menschen sind u. a. der erschwerte Zugang zu den Adressaten, die Vergrößerung gesundheitlicher Ungleichheiten durch Angebote, die die sozialen Lagen nicht berücksichtigen, und Komm-Strukturen sowie die mögliche Diskriminierung, Stigmatisierung oder Schuldzuweisung im Sinne des „blaming the victim“. Das heißt, dass dem Opfer die Schuld für seine Lage zugesprochen wird, obwohl überwiegend strukturelle Bedingungen verantwortlich sind. Problematisch wird auch gesehen, wenn Expertinnen und Experten festlegen, was für sozial benachteiligte Gruppen sinnvoll ist, ohne diese miteinzubeziehen [22].

**Figure 1:**
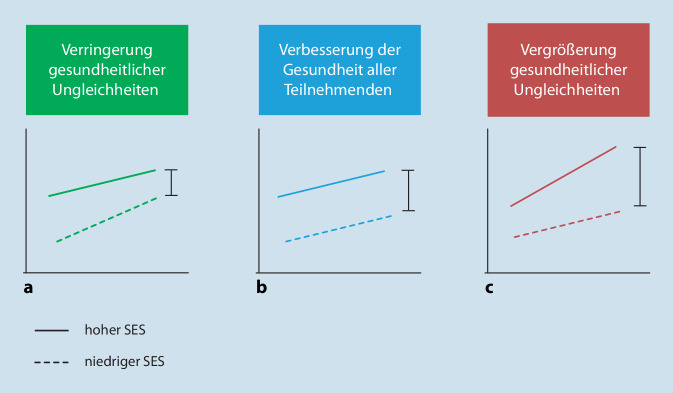

Wenngleich das Ziel von Gesundheitsförderung die Verringerung gesundheitlicher Ungleichheiten ist, können gesundheitsfördernde und präventive Interventionen auch zu einer Vergrößerung dieser Ungleichheiten führen oder keine Effekte aufweisen (Abb. 1). Maßnahmen, die das Potenzial haben, zu einer Verringerung gesundheitlicher Ungleichheiten beizutragen (Abb. 1a), müssen vor allem erreichen, dass sozial Benachteiligte im stärkeren Maß von den Maßnahmen profitieren und damit zu den Privilegierteren gesundheitlich „aufholen“ können [23]. Andererseits ist es ebenso denkbar, dass Maßnahmen zu einer Verbesserung der Gesundheitssituation aller (Teilnehmenden) beitragen, unabhängig von ihrer sozioökonomischen Position. Das ist insgesamt positiv zu werten, führt jedoch auch dazu, dass die gesundheitliche Differenz zwischen sozioökonomisch besser und schlechter gestellten Personen nach wie vor bestehen bleibt (Abb. 1b). In dem dritten Fall können Maßnahmen auch zu einer Vergrößerung gesundheitlicher Ungleichheiten beitragen (Abb. 1c; [23]), wenn sozioökonomisch Benachteiligte nicht oder nicht ausreichend von den Maßnahmen erreicht werden, sie nicht in gleicher Weise von diesen Maßnahmen profitieren oder die Intervention selbst Ungleichheiten verstärkt [21, 24]. Diese unterschiedlichen Entwicklungen ergeben sich beispielsweise durch sozial unterschiedlichen Zugang, Inanspruchnahme und Akzeptanz der Interventionsmaßnahmen, die, so die Kritik, oftmals mittelschichtsorientiert sind und häufiger jene mit höherem Sozialstatus besser erreichen [22]. Das spiegelt sich auch in der *Inverse-Equity-Hypothese* wider, die davon ausgeht, dass privilegierte Gruppen gegenüber nichtprivilegierten allgemeinhin empfänglicher für Gesundheitsförderungsmaßnahmen sind [25].

Bei der Unterscheidung dieser 3 Wirkungsweisen handelt es sich jedoch um eine reduzierte und vereinfachte Darstellung möglicher Auswirkungen von Interventionen, insbesondere da lediglich ein dualer Vergleich zwischen höheren und niedrigen Statusgruppen vorgenommen wird. So weisen Benach et al. [26] auf insgesamt 8 unterschiedliche Szenarien von Interventionseffekten hin, welche die Auswirkungen sowohl auf die Gesundheit der Bevölkerung im Allgemeinen als auch auf gesundheitliche Ungleichheiten skizzieren [26], was die Komplexität der Thematik verdeutlicht.

## Der Beitrag der Schule zur gesundheitlichen Chancengleichheit

In der Lebensphase der Kindheit und Jugend sollten Strategien da ansetzen, wo junge Menschen leben und aufwachsen. Neben der Familie ist die Schule als sekundäre Sozialisationsinstanz einer der wichtigsten institutionellen Kontexte, der ihre psychosoziale und gesundheitsbezogene Entwicklung maßgeblich beeinflusst [27]. Zum einen stellt die Gesundheitserziehung und -bildung von Kindern und Jugendlichen einen gesetzlich bestimmten Auftrag der Schule dar, der aufgrund der Länderhoheit im unterschiedlichen Ausmaß in den Schulgesetzen der Bundesländer verankert ist [28]. Zum anderen ist natürlich die Bildung Hauptaufgabe der Schule und der Bildungserfolg kann als eine wichtige Ressource für die gesundheitliche Entwicklung von Kindern und Jugendlichen angesehen werden [29]. Gelingt beispielsweise Schülerinnen und Schülern ein Bildungsaufstieg, so wirkt sich das unabhängig von ihrer sozialen Herkunft positiv auf die Gesundheit aus. Und mehr noch, sie können damit sogar ihre Nachteile gegenüber Gleichaltrigen reduzieren, die bereits das gleiche Bildungsniveau aufweisen [30].

Die Schule kann für junge Menschen an sich sowohl einen positiven als auch negativen Einfluss auf die Gesundheit und das Wohlbefinden haben. Protektiv haben sich u. a. eine positive Wahrnehmung der Schule, ein Gefühl der Verbundenheit oder auch die soziale Unterstützung durch Klassenkameradinnen und Klassenkameraden oder durch Lehrkräfte erwiesen. Leistungsdruck, Überforderung im Unterricht, Versagensängste und Mobbing in der Schule, um nur einige Aspekte zu nennen, sind mit einem niedrigen Wohlbefinden und (psychischer) Belastung verknüpft [27, 31]. Schule stellt daher ein wichtiges Handlungsfeld für Gesundheitsförderung dar und ist ein zentrales Setting. Erstmals wurde der Begriff Setting (soziales System, Lebenswelt) und der damit verbundene Ansatz in der Ottawa-Charta zur Gesundheitsförderung (1986) aufgeworfen und im Gesetz zur Stärkung der Gesundheitsförderung und Prävention (Präventionsgesetz) im Jahr 2015 als Strategie verankert [32]. Schulische Gesundheitsförderung hat das Ziel, alle Mitglieder der Schule zu befähigen Verantwortung für die eigene Gesundheit sowie die der Mitmenschen zu übernehmen [33]. Es lassen sich insgesamt 3 verschiedene Realisierungsmöglichkeiten der schulischen Gesundheitsförderung unterscheiden. Der 1) verhaltensbasierte Ansatz richtet sich an einzelne Personengruppen (z. B. Schülerinnen und Schüler) und versteht das Setting als einen Ort, die Zielgruppe zu erreichen (Gesundheitsförderung im Setting). Im Gegensatz dazu richtet sich das Konzept 2) „Gesundheitsfördernde Schule“ an alle schulischen Personengruppen (Lehrkräfte, Schulleitungen, nicht unterrichtendes Personal, Erziehungsberechtigte etc.), wobei die Schule als ganzheitliche Organisation gesehen und verhältnisbezogene Strategien angewandt werden. National wie international wird diesem Ansatz eine deutlich langfristigere Erfolgsaussicht zugesprochen als dem verhaltensbasierten Ansatz, welcher oftmals eher kurz-/mittelfristig erfolgreich ist. Seit etwa 15 Jahren hat sich ein neuer Ansatz 3) „Gute Gesunde Schule“ etabliert. Während die beiden ersten Formen als Ausgangspunkt eine gesundheitliche Perspektive einnehmen, wird hier v. a. eine schulpädagogische Sichtweise eingenommen und eine Verknüpfung von Gesundheit und Bildung hergestellt. Der Ansatz richtet sich ebenfalls an alle schulischen Personengruppen und betrachtet die Schule als Institution des Bildungswesens mit ihrem Bildungs- und Erziehungsauftrag [33].

Insgesamt fehlt es jedoch häufig an evidenten Studien und Wissen, warum, wie und unter welchen Umständen Maßnahmen wirken [34]. Schulen als Interventionsort bieten den Vorteil, dass potenziell alle schulpflichtigen Kinder und Jugendlichen, unabhängig von ihrem sozioökonomischen Hintergrund, mittels schulischer Gesundheitsförderungsstrategien erreicht werden könnten [28]. Allerdings werden gesundheitliche Ungleichheiten im Rahmen schulischer Gesundheitsförderung bislang wenig berücksichtigt. Der Abbau sozialer und gesundheitlicher Ungleichheiten steht oftmals nicht im Fokus von Gesundheitsförderungsprogrammen [35]. So werden sozioökonomisch bedingte Ungleichheiten zumeist entweder ausgeblendet oder unzureichend berücksichtigt [34, 36–40], was dazu führen kann, dass die Schülerinnen und Schüler mit niedriger sozioökonomischer Position wenig(er) von den Maßnahmen profitieren [20, 38].

Reviews evaluativer Interventionen zur Gesundheitsförderung bei Heranwachsenden, die hingegen sozioökonomische Ungleichheiten in der Gesundheit und im Gesundheitsverhalten berücksichtigen, sind zumeist breit angelegt und beziehen sich nicht oder nur unzureichend auf schulbasierte Interventionen (vgl. u. a. [40, 41]). Insgesamt fehlt es daher an Studien, die sowohl schulische Interventionen als auch sozioökonomische Ungleichheiten berücksichtigen und Aussagen darüber treffen können, inwieweit diese Maßnahmen zu einer Verringerung gesundheitlicher Ungleichheiten beitragen. Ausnahmen stellen u. a. Hofmann et al. [38] oder auch Moore et al. [42] dar. Die beiden systematischen Reviews identifizierten Interventionen, die unterschiedliche Effekte auf gesundheitliche Ungleichheiten der Schülerinnen und Schüler hatten. So hat Moore et al. [42] 90 Studien inkludiert, wovon lediglich 20 Studien Effekte bezüglich gesundheitlicher Ungleichheiten untersucht haben, hiervon stammten die meisten aus Europa. In 4 dieser Studien führten die Maßnahmen zu einer Vergrößerung der Ungleichheiten, in 6 Studien konnte eine Verringerung erzielt und in weiteren 10 Studien konnte kein unterschiedlicher Effekt nachgewiesen werden. In Anbetracht der Bedeutung der sozialen Determinanten für die Gesundheit ist es jedoch bemerkenswert, dass mehrheitlich keine ungleichheitsbezogenen Evaluationen mit Subgruppenanalysen im Bereich der schulischen Gesundheitsförderung und Prävention vorliegen [43].

## Ansatzpunkte zur Verringerung gesundheitlicher Ungleichheit bzw. zur Erhöhung gesundheitlicher Chancengleichheit

In der Forschung besteht Konsens darüber, dass zur Reduktion gesundheitlicher Ungleichheiten deren *Ursachen* stärker in den Maßnahmen berücksichtigt werden müssen – und mehr noch: die Ursachen der Ursachen. Es reicht nicht aus, die Ursachen der Erkrankung zu betrachten (z. B. mangelnde Bewegung), vielmehr müssen die dahinterliegenden Ursachen (z. B. die unzureichenden Sportangebote, strukturelle Bedingungen) verstanden werden. Damit zeigt sich, dass eine reine Fokussierung auf das Gesundheits- und Risikoverhalten (verhaltensbezogener Ansatz) nicht ausreicht, da diese in die Lebensbedingungen eingebettet sind. Gemeint sind hier die sogenannten Determinanten der Gesundheit [44]. Ein systematisches Review konnte zeigen, dass 50–100 % der Ungleichheiten in der subjektiven Gesundheit durch materielle und strukturelle Lebensbedingungen, psychosoziale Ressourcen und Belastungen sowie das Gesundheits- und Risikoverhalten erklärt werden können [45]. Auch Faktoren der Mesoebene spielen für die Erklärung gesundheitlicher Ungleichheiten eine Rolle. Beispielsweise zeigt ein Review über institutionelle Merkmale der Schule (Meso), dass Schulcharakteristiken (kompositionelle und kontextuelle Merkmale der Schule und der Klasse) neben Bildungsfaktoren (z. B. schulischen Leistungen) die Gesundheit und gesundheitliche Ungleichheiten beeinflussen [46]. Bei dieser Evidenz ist es unklar, warum sich nach wie vor viele Maßnahmen überwiegend (alleine) auf das Gesundheitsverhalten fokussieren, anstatt strukturelle, verhältnisbezogene Ansätze im Sinne von gesundheitsfördernden Schulen zu wählen, die langfristig erfolgreicher sind [33].

Unter Berücksichtigung des Forschungsstands [20, 35, 38, 47–49] lassen sich einige begünstigende Voraussetzungen bzw. Bedingungen formulieren, die zu einer Erhöhung gesundheitlicher Chancengleichheit beitragen können:

Gesundheitliche Ungleichheiten bei Kindern und Jugendlichen können eher mit solchen schulischen Interventionen reduziert werden, die in die *Lebenswelt* der Heranwachsenden integriert werden, ohne aktives Auf-sie-Zugehen bzw. eine bewusste Auswahlentscheidung der Heranwachsenden vorauszusetzen (Settingansatz). Interventionen hingegen, die eine bewusste Auswahlentscheidung der Heranwachsenden voraussetzen, können zu einer Vergrößerung der Ungleichheit führen.

Es hat sich gezeigt, dass *Verhältnisprävention* (strukturelle Ansätze) hinsichtlich der Verringerung gesundheitlicher Ungleichheiten wirksamer zu sein scheint als Verhaltensprävention, bei der v. a. edukative Maßnahmen zu einer Vergrößerung gesundheitlicher Ungleichheiten beitragen können. Kombinierte Ansätze scheinen am erfolgreichsten zur Reduktion gesundheitlicher Ungleichheiten [19, 20, 23].

Auch unterscheidet sich die Wirksamkeit universeller oder selektiver Interventionen in der schulischen Gesundheitsförderung nach der *Art und Qualität der Intervention*. Faktoren, wie z. B. die Dauer der Intervention, deren konsequente Umsetzung, die Integration der Programminhalte in die Curricula sowie das Einbeziehen von Eltern, Lehrkräften und Peers, kommt hier eine entscheidende Rolle zu. Interventionen, die universell ausgelegt sind, können zwar zu einer generellen Verbesserung der Gesundheit führen [50], jedoch können sie gesundheitliche Ungleichheiten auch verstärken [26, 37].

Als wichtiges Instrument zur Erhöhung gesundheitlicher Chancengleichheiten wird die *Partizipation* der Zielgruppe bei der Planung und Implementierung von Maßnahmen gesehen [51]. Pulimeno et al. (2020) plädieren dafür, dass das Schulsystem wie auch die Lehrkräfte vor allem die Stärken (emotionale und soziale) der Kinder und Jugendlichen forcieren sollten – im Sinne einer „warmen Decke der Prävention“ [52, 53]. Damit sollen u. a. junge Menschen zu gesunden Entscheidungen ermutigt und im Sinne des Empowerments in ihrer Autonomie und Selbstbestimmung bestärkt werden [53].

Die genannten Befunde beziehen sich überwiegend auf Studien in anderen Ländern. Inwiefern die Ergebnisse auf Deutschland übertragen werden können, bleibt überwiegend unklar. Selbst innerhalb Deutschlands unterscheiden sich die Schulen in ihren Voraussetzungen und kulturellen Kontexten, sodass Ergebnisse nicht unbedingt für alle Schulen gleichermaßen gelten und daher eine differenzierte Sicht notwendig ist.

### Situation in Deutschland

Die Verbesserung der gesundheitlichen Lage sozial Benachteiligter wird zunehmend auch in Deutschland aufgegriffen, auch wenn die Maßnahmen meist relativ klein, regional und zeitlich begrenzt sind [44]. Einige wichtige Ansätze im bundesweiten Kontext werden im Folgenden vorgestellt.

#### Gesetzliche Verankerung des § 20 Fünftes Buch Sozialgesetzbuch (SGB V).

Eine wichtige Grundlage ist die gesetzliche Verankerung des § 20 SGB V. Die Verringerung gesundheitlicher Ungleichheiten wird damit als Aufgabe der gesetzlichen Krankenversicherungen gesetzlich festgeschrieben. Demnach müssen spezielle Gesundheitsförderungs- und Präventionsmaßnahmen zur Verbesserung der gesundheitlichen Lage bei sozial benachteiligten Personen erfolgen. Der Paragraf wurde bereits im Jahr 2000 aufgenommen, im Präventionsgesetz von 2015 liegt hierzu die aktuelle Fassung vor [44]. Diese Bemühungen spiegeln sich auch im Präventionsbericht der gesetzlichen Krankenkassen wider, die settingbasierte Maßnahmen in „sozialen Brennpunkten“^1^ durchführen. 27 % der Aktivitäten in Grundschulen wurden im Jahr 2020 in diesen sog. sozialen Brennpunkten durchgeführt. Der entsprechende Anteil bei weiterführenden Schulen konnte dem Präventionsbericht nicht entnommen werden [54].

#### Kooperationsverbund gesundheitliche Chancengleichheit.

Der nationale Kooperationsverbund wurde 2003 gegründet und fokussiert sich insbesondere auf die Verbesserung der gesundheitlichen Lage sozial benachteiligter Gruppen. Die Aktivitäten umfassen u. a. 1) die Entwicklung der 12 Kriterien für eine gute Praxis, 2) die Etablierung einer Koordinierungsstelle gesundheitlicher Chancengleichheit in jedem Bundesland, 3) die Erstellung einer Praxisdatenbank zu gesundheitsförderlichen Maßnahmen, die gezielt bei sozial benachteiligten Gruppen angewendet wurden, sowie 4) den Aufbau kommunaler Partnerprozesse hinsichtlich kommunaler Gesundheitsförderung [44]. Ziel der 12 Kriterien guter Praxis war die Entwicklung von Maßnahmen zur Verringerung gesundheitlicher Ungleichheiten beruhend auf der bisherigen wissenschaftlichen Evidenz unter Einbezug der Praxis. Die 12 Kriterien sollen in Settings, z. B. der Schule, umgesetzt werden und umfassen: Zielgruppenbezug, Konzeption der Maßnahme, Settingansatz, Empowerment, Partizipation, niedrigschwellige Arbeitsweise, Multiplikatorenkonzept, Nachhaltigkeit, integriertes Handlungskonzept, Qualitätsmanagement, Dokumentation und Evaluation sowie Wirkungen und Kosten der Maßnahme. Diese Kriterien wurden auch in den „Leitfaden Prävention“ der gesetzlichen Krankenkassen aufgenommen und dienen damit als Qualitätskriterien für Maßnahmen, die von der gesetzlichen Krankenversicherung finanziert werden können [38]. In der Praxisdatenbank des Kooperationsbundes finden sich derzeit fast 700 Projekte im Setting Schule, allerdings erfüllen davon nur 34 Projekte die 12 Kriterien guter Praxis.^2^

#### Initiative des Bundesministeriums für Gesundheit (BMG).

Im Jahr 2019 konzipierte das Bundesministerium für Gesundheit den „Wegeweiser zum Verständnis der Gesundheitsförderung und Prävention“ [55]. Die Initiative geht der Frage der Stärkung und Verbesserung gesundheitlicher Chancengleichheit nach und bindet in ihren Fachdialog Beteiligte aus Wissenschaft, Praxis und Politik, aus den Bereichen Gesundheitswesen, Bildungs‑, Gesundheits- und Sozialwissenschaften, Familien‑, Kinder- und Jugendhilfen sowie den zuständigen Ministerien ein. Der „Wegweiser“ kann als eine Form der Orientierung verstanden werden, dem Stakeholder auf dem Gebiet der Kindergesundheit zentrale Eckpunkte zum Verständnis von Gesundheitsförderung entnehmen können. Es wird ein 10-Punkte-Plan vorgestellt, der explizit auch gesundheitliche Chancengleichheit umfasst. Dieser soll vor allem lebensweltübergreifend und kommunal verankert werden und sowohl auf Empowerment und Kompetenzentwicklung als auch auf aktive Partizipation von Kindern und Jugendlichen abzielen. Die Schule bietet als Lebenswelt die Möglichkeit, eine nachhaltige Gesundheitsförderung von Kindern und Jugendlichen zu implementieren, indem langfristig angelegte und auch strukturelle Voraussetzungen in Anbindung an ein kommunales Gesamtkonzept geschaffen werden können.

## Diskussion und Ausblick

Zusammenfassend kann festgehalten werden, dass Kinder und Jugendliche in der vergleichsweise gesunden Lebensphase der Adoleszenz bereits ungleiche Lebens- und Gesundheitschancen aufweisen, die sich im Laufe ihres Lebens weiter manifestieren und vergrößern können. Obwohl das Ziel der Gesundheitsförderung und Prävention auch explizit gesundheitliche Chancengleichheit einschließt, zeigen sich hier frappierende Forschungsdefizite. Maßnahmen zur Gesundheitsförderung und Prävention berücksichtigen nach wie vor selten explizit sozial benachteiligte Heranwachsende und Evaluationen können kaum sozioökonomisch differenzierte Aussagen zu der Wirksamkeit der Interventionen treffen. Damit bleibt weitgehend unklar, *wie* Maßnahmen zur gesundheitlichen Chancengleichheit ausgestaltet werden müssen, um einen effektiven Beitrag leisten zu können. Die Schule hat ein hohes Potenzial, die Gesundheit aller Schülerinnen und Schüler, insbesondere sozial Benachteiligter, zu fördern, allerdings steht die Verringerung gesundheitlicher Ungleichheiten bislang nicht im Mittelpunkt von schulischen Gesundheitsförderungsprogrammen – zumindest nicht in der Evaluation statusspezifischer Unterschiede in der Wirksamkeit zur Verringerung dieser. In Deutschland ist das Thema gesundheitliche Chancengleichheit zunehmend im Fokus. Doch gesetzliche Zielvereinbarungen müssen sich auch in den Aktivitäten und in den Ergebnissen der Maßnahmen widerspiegeln – hier zeigt sich noch großes Ausbaupotenzial.

Sowohl die WHO als auch der Kooperationsverbund Gesundheitliche Chancengleichheit haben Kriterien definiert, die für eine gelingende (schulische) Gesundheitsförderung und Prävention zur Erhöhung gesundheitlicher Chancengleichheiten förderlich sein können. Dennoch wurde selten explizit hinterfragt, warum welche Interventionen wirken oder nicht wirken. Hier können sogenannte Realist-Reviews Einblicke gewähren, da diese Art der wissenschaftlichen Arbeit nicht untersucht, *ob* eine Intervention wirkt, sondern *wie* und *warum*. Für die schulische Gesundheitsförderung wurde dies in einem Realist-Review überprüft, das aber leider keine Aussagen zu statusspezifischen Aspekten zulässt [56]. Das Forschungsprojekt „Schulische Interventionen der Gesundheitsförderung und Primärprävention zur Reduzierung gesundheitlicher Ungleichheiten im Kindes- und Jugendalter: Ein Realist Review“ (I-GEP), soll einen Beitrag zu diesem Forschungsdefizit leisten [57, 58]. Das Projekt verfolgt das Ziel, Aussagen darüber zu generieren, wie, warum und unter welchen Bedingungen schulische Gesundheitsförderungs- und Primärpräventionsmaßnahmen zu mehr gesundheitlicher Chancengleichheit bei Kindern und Jugendlichen beitragen können. Damit soll ein tieferes Verständnis zu Wirkungsmechanismen komplexer Interventionen im schulischen Setting unter Einbezug unterschiedlicher Kontexte und Zielgruppen erlangt werden. Die Ergebnisse sollen Stakeholder aus Politik und Praxis in der Ausgestaltung schulischer Gesundheitsförderungsprogramme unterstützen, die zu einer Verringerung gesundheitlicher Ungleichheiten beitragen können. Die Ergebnisse werden Ende 2022 erwartet.

Schulen haben das Potenzial, gesundheitliche Ungleichheiten zu verringern und damit als „ladder out of poverty“ [59] zu fungieren, wenn dieses Potenzial im vollen Umfang ausgeschöpft wird. Dennoch sollte hinterfragt werden, ob den Schulen damit nicht zu viel zugemutet wird bei gleichzeitig knappen zeitlichen und personellen Ressourcen. Die weiterführende Diskussion müsste folglich auch dazu geführt werden, ob die Problemlagen und Ursachen sozialer Ungleichheit nicht stärker in den Blick genommen werden sollten, als – provokant formuliert – diese Handlungsfelder auf die Schulen „abzuwälzen“. Gerade die COVID-19-Pandemie hat die Schulen noch einmal zusätzlich vor Herausforderungen gestellt und soziale Ungleichheiten durch Distanzunterricht, fehlende Digitalisierung und unzureichende Auffangmöglichkeiten weiterhin verschärft [60, 61]. Schließlich können Schulen alleine gesundheitliche Ungleichheiten nicht reduzieren, sie können aber einen maßgeblichen Beitrag dazu leisten: „Schools have the potential to play an important part in improving the education, health and well-being of all young people and in the task of reducing inequalities in health in Europe and across the world“ [62]. Dennoch braucht es einen viel umfassenderen Ansatz, wie wir es bei dem Health-in-all-Policies-Ansatz oder auch hinsichtlich der Präventionsketten sehen. Es wird jedoch Zeit benötigen, bis sich die Investitionen auszahlen. Umso früher die Investition beginnt und umso mehr sich die Bemühungen auf möglichst viele wichtige Lebenswelten erstrecken, die auch ineinandergreifen – sei es in der Schule, im Kindergarten, in der Vorschule oder schon in der Familie –, desto höher sind die Erfolgschancen bei der Verringerung gesundheitlicher Ungleichheiten, die ihrerseits zu einer besseren Gesundheit, mehr sozialer Gerechtigkeit und zur Erhöhung der Wirtschaftlichkeit beiträgt [63].

## Footnotes

1 „Soziale Brennpunkte“: Wohngebiete, die stark von Einkommensarmut, Integrationsproblemen und Arbeitslosigkeit betroffen sind und deren Anwohnende ein begrenztes soziales Netzwerk aufweisen.

2 Siehe Website: https://www.gesundheitliche-chancengleichheit.de/praxisdatenbank; Zugriff: 12.04.2022.
